# Molecular Changes in Tissue Proteome during Prostate Cancer Development: Proof-of-Principle Investigation

**DOI:** 10.3390/diagnostics10090655

**Published:** 2020-08-31

**Authors:** Agnieszka Latosinska, Katarina Davalieva, Manousos Makridakis, William Mullen, Joost P. Schanstra, Antonia Vlahou, Harald Mischak, Maria Frantzi

**Affiliations:** 1 Mosaiques Diagnostics GmbH, 30659 Hannover, Germany; mischak@mosaiques-diagnostics.com (H.M.); frantzi@mosaiques-diagnostics.com (M.F.); 2 Research Centre for Genetic Engineering and Biotechnology “Georgi D Efremov”, Macedonian Academy of Sciences and Arts, 1000 Skopje, North Macedonia; katarina@manu.edu.mk; 3 Biomedical Research Foundation of the Academy of Athens, Centre of Systems Biology, 11527 Athens, Greece; mmakrid@bioacademy.gr (M.M.); vlahoua@bioacademy.gr (A.V.); 4 Institute of Cardiovascular and Medical Sciences, University of Glasgow, Glasgow G12 8QQ, UK; William.Mullen@glasgow.ac.uk; 5 Institut National de la Santé et de la Recherche Médicale (INSERM), U1048, Institute of Cardiovascular and Metabolic Diseases, 31432 Toulouse, France; joost-peter.schanstra@inserm.fr; 6 Université Toulouse III Paul-Sabatier, 31400 Toulouse, France

**Keywords:** drug target, personalized medicine, prostate cancer, proteomics, tissue

## Abstract

(1) Background: Prostate cancer (PCa) is characterized by high heterogeneity. The aim of this study was to investigate molecular alterations underlying PCa development based on proteomics data. (2) Methods: Liquid chromatography coupled to tandem mass spectrometry was conducted for 22 fresh-frozen tissue specimens from patients with benign prostatic hyperplasia (BPH, *n* = 5) and PCa (*n* = 17). Mann Whitney test was used to define significant differences between the two groups. Association of protein abundance with PCa progression was evaluated using Spearman correlation, followed by verification through investigating the Prostate Cancer Transcriptome Atlas. Functional enrichment and interactome analysis were carried out using Metascape and String. (3) Results: Proteomics analysis identified 1433 proteins, including 145 proteins as differentially abundant between patients with PCa and BPH. In silico analysis revealed alterations in several pathways and hallmarks implicated in metabolism and signalling, represented by 67 proteins. Among the latter, 21 proteins were correlated with PCa progression at both the protein and mRNA levels. Interactome analysis of these 21 proteins predicted interactions between Myc proto-oncogene (MYC) targets, protein processing in the endoplasmic reticulum, and oxidative phosphorylation, with MYC targets having a central role. (4) Conclusions: Tissue proteomics allowed for characterization of proteins and pathways consistently affected during PCa development. Further validation of these findings is required.

## 1. Introduction

With more than 1.2 million new cases and almost 360,000 disease-related deaths among men in 2018, prostate cancer (PCa) has contributed to 13.5% of the diagnosed cancer cases and 6.7% of cancer-associated deaths in men [1]. The notable difference between incidence and mortality is related to the fact that significant proportion of prostate tumours are slow-growing, organ-confined tumours that are not likely to progress even without treatment [2]. However, some patients experience an aggressive disease course (life-threatening) that requires immediate therapy [2]. Accurate discrimination between these two phenotypes is a well-recognized clinical challenge [2]. As a result, both, overtreatment of patients with low-risk disease and undertreatment of patients experiencing the aggressive disease are frequently observed [3]. Beyond this aspect, undertreatment also arises from the lack of effective therapies for advanced disease. Although the 5-year survival is close to 100% for patients with cancer confined to the prostate, for patients presenting with distant metastasis, the prognosis is poor, with the 5-year survival rate dropping to around 30% [4]. This indicates that PCa-related deaths are the result of cancer progression and development of metastatic castration-resistant PCa (mCRPC), with a median survival of 2 to 3 years [5].

Advancements in knowledge on the molecular background of PCa indicated high complexity and heterogeneity of the disease [6,7,8]. Even though, over the last decade, new therapies have been approved in the context of castration-resistant PCa (CRPC) and/or mCRPC [9,10], the low response rate and the development of resistance as a result of the diverse molecular background of the tumour stands in the way of improving outcomes. To close this gap, a potential solution may be the global analysis of tumour samples at the molecular level through advanced –omics approaches to better understand the mechanisms underlying disease development and to define an array of drug targets and potential biomarkers to stratify patients for a specific treatment [11]. Among the –omics approaches, investigation of proteins is expected to provide particularly relevant insights into cancer biology, as proteins integrate genomic information with environmental impact, regulate all biological functions and display information on specific disease-altered pathways.

The purpose of this study was to investigate proteomics profiles from malignant and benign prostate tissue to provide insights into molecular pathophysiology underlying PCa development. The findings of this proof-of-concept study are expected to build a solid foundation for identification of drug targets and drug candidates for PCa relying on molecular pathophysiology.

## 2. Materials and Methods 

### 2.1. Clinical Material

We analysed 22 prostate surgical specimens from patients with clinically and histologically confirmed PCa and benign prostatic hyperplasia (BPH) obtained from the University Clinic for Urology, University Clinical Centre “Mother Theresa”, Skopje, Republic of North Macedonia. Informed consent for the use of these samples for research purposes was obtained from the patients in accordance with the Declaration of Helsinki. All data were anonymised, and the study was approved by the ethics committees of the Macedonian Academy of Sciences and Arts (09-1221/1, 10-04-2018) and by the Hannover Medical School (7975_BO_K_2018, 12-07-2018). The samples used in this study were fresh surgical tissues from 5 BPH patients obtained by transurethral resection of the prostate gland and from 17 PCa patients obtained by radical prostatectomy (Table S1). All tissue samples were kept on ice immediately after surgery to avoid proteolytic degradation and were subsequently snap frozen in liquid nitrogen within 30 min after surgery. Samples were stored at −80 °C. Frozen sections were cut from the macroscopically visible tumour areas to confirm histologically the presence of cancer. Manual microdissection was conducted to obtain pathologically characterized materials for our proteomics approach. All tumour samples contained more than 70% of tumour cells.

### 2.2. Sample Preparation and Liquid Chromatography Coupled to Tandem Mass Spectrometry (LC-MS/MS) Analysis

Sample preparation was performed as described in [12,13,14]. In brief, tissue samples (with net weight of 10–20 mg) were homogenized with a bullet blender homogenizer (Next Advance, Troy, NY, US) using stainless steel beads (0.9–2-mm diameter). Homogenization was performed in lysis buffer comprised of 4% sodium dodecyl sulphate (SDS), 0.1 M dithioerythritol and 0.1 M Tris-HCl pH 7.6. Subsequently, the samples were centrifuged at 16,000*g* for 10 min at room temperature and the supernatant was kept in clean tubes. Protein concentration was measured with Bradford assay. Protease inhibitors (Roche, Basel, Switzerland) were added at a final concentration of 3.6% *v*/*v*, and the samples were stored at −80 °C until further use. Ten micrograms of each sample were loaded onto sodium dodecyl sulphate–polyacrylamide gel electrophoresis (SDS-PAGE). Protein separation and tryptic digestion were performed as described in [12,13,14]. Tryptic digests were dried, resuspended in mobile phase A buffer (0.1% formic acid, pH 3) and processed with LC-MS/MS analysis, as previously described [13,14]. Briefly, the peptide mixture was first loaded into a Dionex Ultimate 3000 RSLS nano flow system (Dionex, Camberly, UK), including an integrated Dionex nano trap column (0.1 × 20 mm, 5 µm, C18). Two percent acetonitrile/0.1% formic acid was applied as a mobile phase, and the flow rate was set to 5 µL/min. Acclaim PepMap C18 nano column (75 μm × 50 cm, 2 μm 100 Å) was configured as an analytical column, with a flow rate of 300 nL/min. Peptides were eluted with a gradient of mobile phase A (0.1% formic acid in water) versus mobile phase B (0.1% formic acid in 80% acetonitrile) as follows: 1% B for 5 min rising to 5% B at 10 min and then to 25% B at 360 min and 65% B at 480 min. Electrospray ionisation was applied using a Proxeon nano spray source (positive ion mode) connected to an Orbitrap LTQ Velos (Thermo Finnigan, Bremen, Germany). The instrument was operated in MS/MS mode with the scanning range set at 380 to 2000 *m*/*z*. An ionization voltage of 2.6 kV and temperature of the capillary at 275 °C were applied, while the resolutions of ions were 60,000 and 7500 in MS1 and MS2 (for higher-energy collisional dissociation, HCD), respectively. The top 20 method was implemented for selection of the precursor ions from each scan, followed by fragmentation using HCD at 35% collision energy. Dynamic exclusion was applied with a repeat count of 1 and an exclusion duration time of 30 s.

### 2.3. MS Data Processing and Statistical Analysis

RAW mass spectrometry data were processed and analysed as follows [14,15,16]: Protein identification was conducted using Proteome Discoverer v1.4 (Thermo Finnigan, San Jose, CA, US) using the human SwissProt database and the SEQUEST search engine. The FASTA file was downloaded from the Uniprot database (https://www.uniprot.org/) on 20 June 2019 and included 20,431 reviewed entries. Only canonical sequences were considered. The following search parameters were applied: (a) precursor mass tolerance: 10 ppm and fragment mass tolerance: 0.05 Da; (b) full tryptic digestion; (c) maximum missed cleavage sites: 2; (d) static modifications: carbamidomethylation of cysteine; and (e) dynamic modifications: oxidation of methionine and proline, deamidation of asparagine and glutamine, carbamylation of lysine and N-terminal carbamidomethylation. Subsequently, individual datasets were exported at the peptide level using the following filters: (a) peptide confidence: high, medium and low; (b) peptide rank up to 5; (c) peptide grouping enabled and protein grouping disabled; and (d) ∆M ± 5 ppm. Data were further processed using a clustering approach that was described previously, with some minor modifications [15,16]: peptides from different proteomics analyses were grouped (“clustered”) based on a predefined mass window of ±5 ppm and retention time of ±5%. To increase the validity of the reference sequence assignment to the cluster, only sequences that were determined with high confidence during Proteome Discoverer analysis (false discovery rate, FDR < 1%) were considered. Among them, the sequence with the highest sum of Xcorr across all samples was accepted as representative for the cluster. This resulted in the generation of the common peptide list based on all proteomics runs (dataspace) that was subsequently used for protein identification. For protein identification, when the peptide corresponded to multiple proteins, it was assigned only to one protein based on Occam’s Razor approach (i.e., protein that was identified with the highest number of peptides in our dataspace). All identified proteins (independent from the number of peptide–spectrum matches and assigned peptides) were considered for quantification and normalization. Briefly, quantification of proteomics data was based on the precursor ion peak area. Abundance for a given protein was calculated based on the sum of all belonging peptide peak areas [15,16], followed by part per million (ppm) normalization. Statistical analysis was conducted using Mann Whitney test, and differences in protein abundance with *p <* 0.05 were defined as significant. The Spearman’s rank-order correlation was employed to identify proteins associated with PCa progression. For the latter, the protein abundance was correlated with Gleason score (GS) (GS < 7, GS = 7 and GS > 7) from patients with PCa.

### 2.4. Bioinformatics Analysis

Functional enrichment analysis for Molecular Signatures Database (MSigDB) hallmark gene set collection associations was performed using Metascape (https://metascape.org/ [17]). Default settings were applied (hypergeometric *p <* 0.01, ≥3 molecules assigned and an enrichment factor >1.5). Enrichment in the Kyoto encyclopedia of genes and genomes (Kegg) pathways [18] was performed using String v11.0 (https://string-db.org/) [19]. The latter tool was also used to create protein–protein interaction networks. In both cases, default settings were applied [19]. Protein class was assigned based on the Panther Classification System (http://www.pantherdb.org/ [20]).

### 2.5. Transcriptomics Analysis

Transcriptome data were retrieved from the Prostate Cancer Transcriptome Atlas (http://www.thepcta.org/, v1.0.1) [21], comprised of 1321 clinical specimens from 38 PCa cohorts. One-way ANOVA testing was used to detect associations with disease course/progression. Disease progression was defined across the following subgroups: benign, GS < 7, GS = 7, GS > 7 and mCRPC.

## 3. Results

### 3.1. Proteomics Analysis

Tissue proteomics profiling data were acquired from 22 fresh-frozen tissue samples including 17 from patients diagnosed with PCa (*n* = 5 GS6, *n* = 5 GS7 (3 + 4), *n* = 2 GS8 and *n* = 5 GS9) and 5 from patients with BPH. There was no significant difference in age between patients groups (*p* = 0.7243), with median ages of 66 (Interquartile Range (IR): 64.75–71.50) and 66 (IR: 63.50–76.50) for patients with PCa and BPH, respectively, whereas the median prostate-specific antigen (PSA) levels differed significantly (PSA level of 19.9 ng/mL (IR: 13.38–39.35) and 0.9 ng/mL (IR: 0.78–1.58) for patients with cancer diagnosis and benign pathology, respectively, *p* = 0.0009). Clinical and demographic data from the study cohort are presented in Table S1.

**Figure 1:**
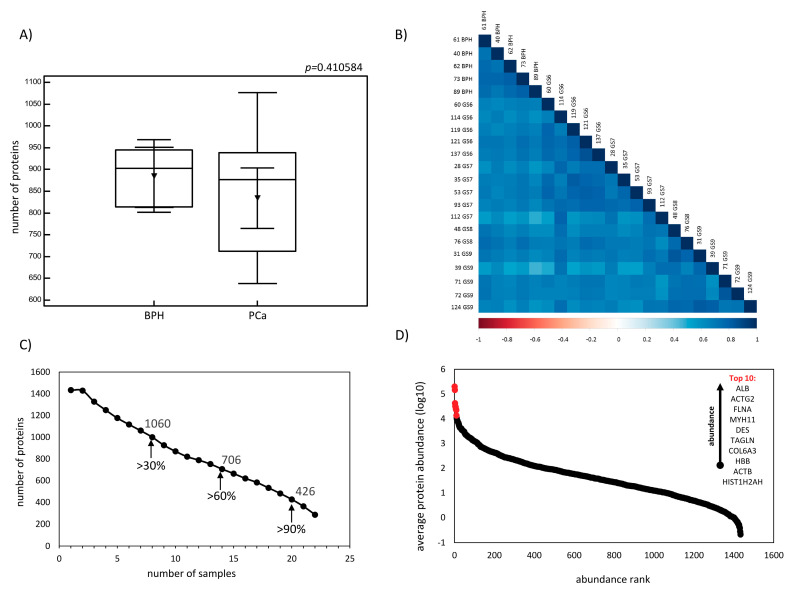
Tissue proteome characterization: (**A**) Boxplots representing the number of proteins identified in prostate tissue samples from patients with benign prostatic hyperplasia (BPH) and prostate cancer (PCa). (**B**) Graphical representation of the correlation matrix for normalized protein abundances across the individual samples: the Spearman Rho correlation coefficient is colour coded. All relationships were significant at *p* < 0.05. (**C**) The data completeness plot reflects representation of the number of proteins identified in the specific number of samples. The number of proteins identified in more than 30%, 60% and 90% of samples are indicated. (**D**) Graphical representation of protein rank against the average protein abundance (log10) calculated based on all analysed samples (*n* = 22): Ten proteins with the highest abundance are highlighted in red. Abbreviations: ALB—serum albumin, ACTB—actin, cytoplasmic 1, ACTG2—actin, gamma-enteric smooth muscle, BPH—benign prostatic hyperplasia, COL6A3—collagen alpha-3(VI) chain, DES—desmin, FLNA—filamin-A, HBB—haemoglobin subunit beta, HIST1H2AH—histone H2A type 1-H, MYH11—myosin-11, PCa—prostate cancer and TAGLN—transgelin.

Following strict criteria for data processing, proteome analysis resulted in the identification of 1433 proteins (on average, 846 protein identifications per sample). A comparable number of proteins was identified in both groups (*p* = 0.4106, Kruskal–Wallis test), with an average of 885 and 834 identified proteins in patients with BPH and PCa, respectively (Figure 1A). In addition, there was a significant correlation (*p* < 0.05) in protein abundance across individual samples (Figure 1B), with a median Spearman Rho correlation coefficient of 0.68 (IR: 0.65–0.74). A total of 1060 (out of 1433) proteins was identified in >30% of samples (Figure 1C). Serum albumin (ALB), actin, gamma-enteric smooth muscle (ACTG2), filamin-A (FLNA), myosin-11 (MYH11), desmin (DES), transgelin (TAGLN), collagen alpha-3(VI) chain (COL6A3), haemoglobin subunit beta (HBB), actin, cytoplasmic 1 (ACTB) and histone H2A type 1-H (HIST1H2AH) were among top 10 highly abundant proteins in the dataset (Figure 1D).

### 3.2. Differences in Protein Abundance between Patients with PCa and BPH

**Figure 2:**
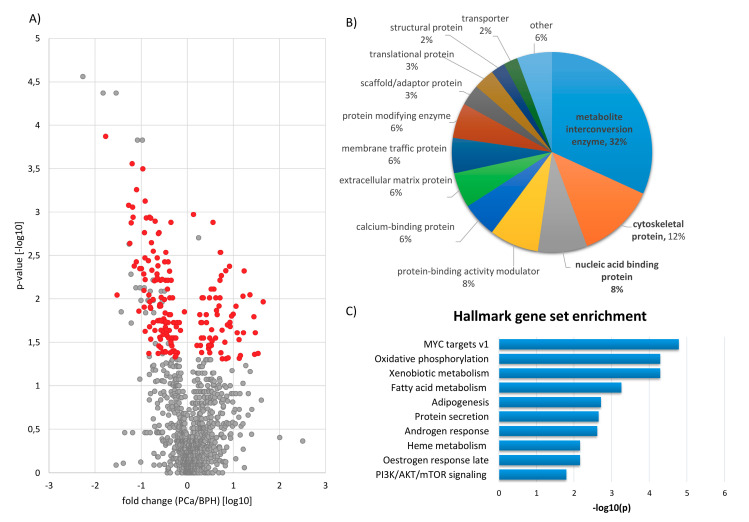
Protein differences between patients with PCa and BPH: (**A**) Volcano plot. Proteins that were identified only in one of the two groups were not plotted. The latter covers 217 and 50 proteins found solely in the case (PCa) and the control (BPH) groups, respectively. Differentially abundant proteins (significant change in the abundance (*p* < 0.05), detected in more than 30% of samples) are shown in red. (**B**) Distribution of protein classes of differentially expressed proteins: Protein classes were defined according to the Panther Classification System. Information on the protein class was available for 88 out of 145 differentially abundant proteins. (**C**) Graphical representation of the enrichment analysis based on Molecular Signatures Database (MSigDB) hallmark gene set ontology: the ten most significantly enriched terms are presented. *p*-values were calculated using the Banjamini–Hochberg procedure. Abbreviations: BPH—benign prostatic hyperplasia, MSigDB—Molecular Signatures Database, MYC—Myc proto-oncogene and PCa—prostate cancer.

A total of 276 proteins was found to be significantly altered (*p* < 0.05, Mann Whitney test) between samples from patients with PCa and BPH (Figure 2A). To increase confidence in the detected differences, only proteins detected in >30% of the samples were considered. Following these criteria and upon removing proteins originating from blood/plasma, 145 proteins (defined for the purpose of this manuscript as “PCa signature”) were considered for subsequent evaluation (Figure 2A). The latter included 72 and 73 proteins being up- and downregulated in cancer in comparison to benign tissue, respectively. The list of differentially abundant proteins is presented in Table S2.

To link differentially abundant proteins to disease pathophysiology, bioinformatics analysis was conducted. The top 3 protein classes represented in the PCa signature included metabolic interconversion enzymes, cytoskeletal proteins and nucleic acid binding proteins (Figure 2B). Proteins belonging to metabolic interconversion enzymes included (among others) dehydrogenases (including retinal dehydrogenase 2 (ALDH1A2), NADH-ubiquinone oxidoreductase 75 kDa subunit (NDUFS1), aldehyde dehydrogenase X (ALDH1B1), succinate (SDHA), 6-phosphogluconate (PGD), sorbitol (SORD) and methylmalonate-semialdehyde (ALDH6A1) dehydrogenases, and glyoxylate reductase/hydroxypyruvate reductase (GRHPR)); peroxidases (e.g., catalase (CAT), peroxiredoxin-6 (PRDX6) and phospholipid hydroperoxide glutathione peroxidase (GPX4)); oxidases (e.g., cytochrome c oxidase subunit 5B (COX5B)), hydratases (aconitate hydratase (ACO2) and bifunctional purine biosynthesis protein ATIC (ATIC)) and others. Examples of cytoskeletal proteins included PDZ and LIM domain protein 5 (PDLIM5), coronin-1B (CORO1B), cysteine and glycine-rich protein 2 (CSRP2), whereas among nucleic acid binding proteins were poly(rC)-binding protein 1 (PCBP1), small nuclear ribonucleoprotein Sm D3 (SNRPD3) and interleukin enhancer-binding factor 3 (ILF3).

Consistent with the above observations, the Kegg pathway enrichment analysis (Table 1) demonstrated that most of the significantly enriched pathways were related to metabolism (e.g., metabolic pathways, tricarboxylic acid (TCA) cycle, carbon metabolism, glutathione metabolism, glyoxylate and dicarboxylate metabolism, pentose and glucuronate interconversions). Other enriched pathways included protein processing in endoplasmic reticulum (ER), interleukin 17 (IL-17) signalling pathways and lysosomes. The enrichment in MSigDB hallmark gene sets (Table 2, Figure 2C) revealed associations with oxidative phosphorylation, xenobiotic metabolism, fatty acid metabolism, adipogenesis, heme metabolism or protein secretion, in line with the enrichment analysis based on Kegg. Other hallmarks (not covered by Kegg analysis) included Myc proto-oncogene (MYC) targets, androgen response, oestrogen response late and PI3K/AKT/mTOR signalling.

### 3.3. Association of Protein Abundance with PCa Progression

Significantly enriched Kegg pathways (Table 1) and MSigDB hallmarks (Table 2) were represented by a total of 67 proteins, with most proteins that act in metabolic pathways identified with higher expression in malignant compared to nonmalignant prostate tissue. For those 67 proteins, association with cancer progression (as represented by increased GS) was investigated by Spearman correlation. Significant correlation of protein abundance with progression was observed for 25 proteins (i.e., 23 positively correlated and 2 negatively correlated; Table 3). In most cases, the direction of the association was in line with the direction of the fold change when comparing protein abundance between patients with PCa and BPH, with the exception of aldo-keto reductase family 1 member B1 (AKR1B1), carbonic anhydrase 2 (CA2) and acetyl-CoA carboxylase 1 (ACACA). These three proteins showed significantly lower abundance in cancer in comparison to controls (*p* < 0.05), while their abundance was positively correlated with GS (*p* < 0.05). For the shortlisted 25 proteins, association with disease progression was further evaluated in an independent transcriptomics dataset available from the Prostate Cancer Transcriptome Atlas [21]. The significant association with disease progression at the mRNA level was found for 21 out of the 25 proteins (*p* < 0.05, ANOVA, Table 3), supporting validity of the proteomics findings. 

**Figure 3:**
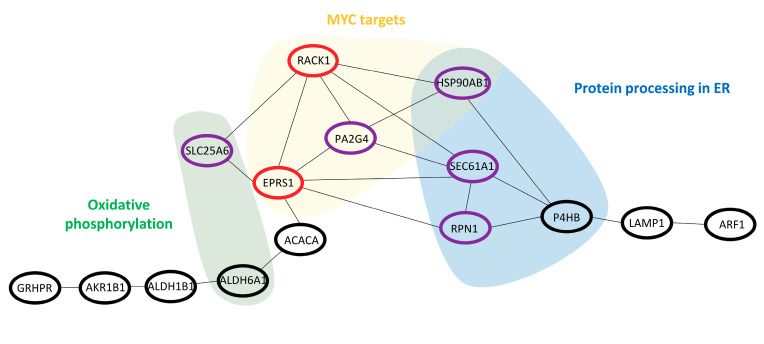
Protein–protein interaction network and associated pathways/hallmarks: Interaction network of proteins associated with progression at protein and mRNA levels. Nodes (proteins) connecting all three pathways/hallmarks are highlighted in red, while those nodes connecting between two pathways/hallmarks are marked in purple. Disconnected nodes in the network are not shown. Colour clouds represent proteins belonging to the indicated pathway/hallmark. Abbreviations: ACACA—acetyl-CoA carboxylase 1, ALDH1B1—aldehyde dehydrogenase X, mitochondrial, ALDH6A1—methylmalonate-semialdehyde dehydrogenase (acylating), AKR1B1—aldo-keto reductase family 1 member B1, ARF1—ADP-ribosylation factor 1, EPRS1—bifunctional glutamate/proline–tRNA ligase, ER—endoplasmic reticulum, GRHPR—glyoxylate reductase/hydroxypyruvate reductase, HSP90AB1—heat shock protein HSP 90-beta, LAMP1—lysosome-associated membrane glycoprotein 1, MYC— Myc proto-oncogene, P4HB—protein disulphide-isomerase, PA2G4—proliferation-associated protein 2G4, RACK1—receptor of activated protein C kinase 1, RPN1—dolichyl-diphosphooligosaccharide–protein glycosyltransferase subunit 1, SEC61A1—protein transport protein Sec61 subunit alpha isoform 1 and SLC25A6—ADP/ATP translocase 3.

**Figure 4:**
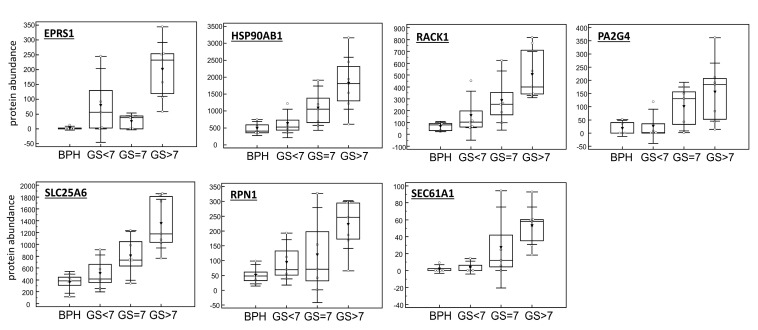
Boxplots representing distribution of the normalized protein abundance according to the disease progression. Abbreviations: BPH—benign prostatic hyperplasia, EPRS1—bifunctional glutamate/proline–tRNA ligase, GS—Gleason score, HSP90AB1—heat shock protein HSP 90-beta, PA2G4—proliferation-associated protein 2G4, RACK1—receptor of activated protein C kinase 1, RPN1—dolichyl-diphosphooligosaccharide–protein glycosyltransferase subunit 1, SEC61A1—protein transport protein Sec61 subunit alpha isoform 1 and SLC25A6—ADP/ATP translocase 3.

To find connections between these 21 proteins and to identify pathways/hallmarks that are consistently affected during disease development, protein–protein interaction analysis was conducted. As a result of this analysis, an interactome network comprised of 15 interconnected proteins with a total number of 22 edges and a significant protein–protein interaction enrichment *p*-value (*p* = 0.000167) was constructed (Figure 3). The latter indicates that proteins included in the network reveal a significantly higher number of interactions between each other than expected when analysing a random protein list of comparable size. This significant enrichment implies that the proteins belonging to the network are likely biologically connected. To further investigate their link to biology, pathways/hallmarks in which these proteins act were overlaid on the interactome network (Figure 3). Interconnection was observed between three pathways/hallmarks, i.e., MYC targets, oxidative phosphorylation and protein processing in ER. Among the interacting proteins involved in these pathways/hallmarks, receptor of activated protein C kinase 1 (RACK1) and bifunctional glutamate/proline–tRNA ligase (EPRS1), both being MYC targets, appear to be connecting nodes with proteins belonging to protein processing in ER and oxidative phosphorylation, while ADP/ATP translocase 3 (SLC25A6), protein transport protein Sec61 alpha isoform 1 (SEC61A1), dolichyl-diphosphooligosaccharide-protein glycosyltransferase subunit 1 (RPN1) and proliferation-associated protein 2G4 (PA2G4) are connecting nodes with only one other pathway/hallmark. Heat shock protein HSP 90-beta (HSP90AB1, HSP 90) is a member of protein processing in ER as well as a MYC target. Distribution of the protein abundance for these proteins, representing connecting nodes, is shown in Figure 4.

## 4. Discussion

This proof-of-concept study aimed to characterize protein changes in tissue that occur during PCa development and to identify affected molecular pathways. A high number of proteins could be identified as significantly affected, many of these in agreement with reports on the transcriptome level. In silico analysis of proteins differentially abundant between PCa and BPH clearly indicated that the vast majority of pathways were related to metabolism. Metabolic reprograming is one of the main cancer hallmarks [22], and its role in the PCa initiation, progression and resistance to therapies has been extensively studied [23,24,25]. Further investigation of the individual metabolic pathways revealed that many of them are well-established hallmarks of PCa, including, among others, fatty acid metabolism, TCA cycle and oxidative phosphorylation. Fatty acid metabolism has been recognized as a dominant process responsible for energy production in PCa cells due to slow glycolysis [26]. Fatty acid synthesis is required for energy production, membrane synthesis and posttranslational modifications [27]. Along these lines, previous studies have demonstrated also acceleration of the TCA cycle in the context of PCa [25], representing one of the key features of prostate malignant transformation. In contrast, normal prostate epithelial cells are specialized to produce and secrete citrate, a component of prostatic fluid, due to inhibition of the TCA cycle. In concordance with the activation of TCA cycle, our analysis revealed also an activation of oxidative phosphorylation. In comparison to other malignancies, PCa depends more on oxidative phosphorylation instead of glycolysis [25,28]. In addition to the metabolic changes, other pathways and biological functions that have been previously linked to PCa were also predicted based on proteomics analysis. Among others, IL-17 signalling [29], protein processing in ER [30,31], androgen signalling [32], PI3K/AKT/mTOR signalling [33] or MYC targets [34] were significantly enriched based on the proteomic PCa signature. The role of the most promising pathways is further discussed below. The identification of pathways/hallmarks that have been previously reported in the context of PCa serves as a positive control for our study and supports its validity. 

To date, numerous molecular subtypes have been defined for PCa [6,7,8]. Thus, classification of samples based on clinical characteristics might not be optimal, also reflected by the moderate success of treatment. Taking this into consideration, we investigated PCa progression (expressed as increase in GS) based on the hypothesis that cancer progresses as a continuum and that proteins that are truly associated with this process are gradually and consistently changed. To further enhance the validity of the findings, the association with disease progression was also assessed at the mRNA level. This complementary assessment through cross-correlation of different –omics traits is expected to better reflect the clinical reality and to increase the validity of individual observations. The approach of cross-correlating proteomics and transcriptomics data has previously shown increased validity [35]. Such cross-omics analysis followed by interactome analysis highlighted prominent alterations in MYC targets, oxidative phosphorylation and protein processing in the ER. Interestingly, these processes seem to be interconnected, with MYC targets linking the latter two pathways. These pathways/hallmarks appear activated during PCa development, and abundance of the associated proteins (also verified at the mRNA level) was found to increase along with disease progression. Multiple evidence to support the implication of these pathways/hallmarks in the context of PCa, along with their interconnections, was collected, as discussed below.

MYC is a proto-oncogene frequently overexpressed in PCa [36] and associated with PCa progression [37]. In principle, MYC increases the expression of genes involved in cell growth and proliferation, survival of cancer cells [37,38] and regulation of cellular metabolism [39]. It promotes transcription of androgen receptor (AR) and enhances the stability of full-length AR and its splice variants [40]. Our study revealed several MYC targets being consistently upregulated during PCa development, including RACK1, HSP 90, EPRS1, and PA2G4. RACK1 [41], HSP 90 [42] and PA2G4 [43] have been reported previously in the context of PCa. RACK1 is a scaffolding protein involved in recruitment, assembly as well as regulation of signalling molecules and has been observed to promote proliferation, invasion and metastasis of PCa both in vitro and in vivo [41]. The molecular chaperone HSP 90 is involved in protein folding and maintaining protein stability (including AR) and has been reported to be overexpressed in many cancers, including PCa [42]. Considering its interactions with AR, HSP 90 was proposed as a potential therapeutic target in PCa [42]. PA2G4 is involved in cellular proliferation and regulation of gene expression and is also a corepressor of the AR. Significant increase in the protein expression from normal to hormone refractory PCa was reported previously, also correlating with the nuclear expression of AR in normal adjacent and cancer tissue [43].

Our analysis showed a connection between MYC targets and protein processing in ER. In fact, activation of ER stress/the unfolded protein response has been linked to cancer, including also PCa [31]. The unfolded protein response represents protective mechanisms of cancer cells under unfavourable conditions (e.g., hypoxia, oxidative stress and eventually ER stress). During the unfolded protein response, inositol requiring-enzyme 1 alpha (IRE1α)/X-box-binding protein 1 (XBP1) signalling is activated [31]. It has been shown that androgens regulate expression of genes associated with ER stress, including the IRE1α-XBP1 arm [31]. Among the proteins contributing to the interactome network described in our study and associated with protein processing in ER, we found SEC61A1 (SEC61 translocon subunit), a component of the protein translocation machinery (together with SEC62 and SEC63) mediating transport across ER [44]. SEC61A1 is a downstream XBP1 target gene, downregulated upon inhibition of XBP1 splicing with MKC-3946 treatment in multiple myeloma cells [45]. Nevertheless, the role of SEC61A1 has not been investigated in the context of PCa. Another component of the ER translocation machinery (although not found in our proteomics analysis), SEC62, has been investigated in PCa. SEC62 knockdown reduced the migration and invasion of PCa cells [46], and the overproduction of SEC62 protein in PCa tissue was correlated with GS [46]. In addition, our analysis identified RPN1, one of the subunits of the oligosaccharyl transferase complex, also being associated with the SEC61 complex. RPN1 is involved in N-glycosylation and was found (among other genes) to be downregulated upon treatment of androgen-independent PCa xenografts with 17β oestradiol that led to inhibition of tumour growth [47]. Protein disulphide-isomerase (P4HB) was another protein identified in our network as a member of protein processing in the ER pathway. P4HB is responsible for the formation, opening and reorganization of disulphide bonds. When present in high concentration, it acts as a chaperone that protects from the generation of misfolded protein aggregates. The P4HB gene was found to be significantly increased in PCa in comparison to the normal prostate gland [48], in agreement with our data.

Based on the available literature and in line with our results, protein processing in ER appears to be interconnected with MYC signalling [49]. Treatment with an IRE1α inhibitor inhibited PCa growth in vitro and in vivo, indicating that the IRE1α/XBP1 pathway promotes PCa through activation of c-MYC signalling [50]. Based on these findings, inhibition of IRE1α/XBP1 was proposed as a possible strategy for PCa treatment. In another study, interaction between IRE1α and RACK1 was crucial for the activation of IRE1α/XBP1 signalling upon unfolded protein response induced by sorafenib in hepatocellular carcinoma cells [51], further supporting the interconnection between MYC signalling and ER processes. 

The data presented here also highlighted oxidative phosphorylation as a hallmark in PCa and indicated its possible connection with MYC targets. Changes in mitochondrial metabolism covering oxidative phosphorylation are one of the cancer hallmarks [52]. A recent study demonstrated alterations in oxidative phosphorylation in paired benign and malignant human prostate tissue samples [28], supporting the validity of our findings. Among the proteins mapped to oxidative phosphorylation in our network, ALDH6A1, an enzyme catalysing the oxidative decarboxylation of malonate and methylmalonate semialdehydes to acetyl- and propionyl-CoA, was found to be overexpressed in PCa tissues in comparison to normal prostatic tissue and was also significantly associated with lymphatic invasion in PCa [53]. In addition, SLC25A6, involved in exchanging ADP from cytoplasm with ATP from mitochondria through the mitochondrial membrane, was also found in our analysis among the proteins involved in oxidative phosphorylation. Furthermore, evidence supporting the connection of MYC with oxidative phosphorylation exists. Among others, MYC is known to be involved in the stimulation of mitochondrial biogenesis [54]. Previous transcriptomics analysis revealed several nuclear genes encoding mitochondrial proteins being downstream targets of MYC, including among others proteins involved in oxidative phosphorylation [54], which supports our findings.

Our study presents with some limitations. Since this was a proof-of-concept study, the sample size was not adequate to perform multiple testing correction. Therefore, identification of differentially expressed proteins was based on the unadjusted *p*-values. Even though, our findings were in line with the existing literature and, for several proteins, a consistent association with disease progression was observed at both the protein and mRNA levels. Further validation of the findings in the context of a statistically well-powered study is required.

## 5. Conclusions

Tissue proteome analysis allowed characterization of molecular changes associated with PCa development. Among the most promising pathways and biological functions consistently affected in disease onset and progression were protein processing in ER, oxidative phosphorylation and MYC targets. These pathways also appear to be linked with each other, which is supported by existing literature. However, their interconnections through the proteins identified in our study are mostly novel and require further validation. These molecular linkers may also serve as potential candidates for drug targeting.

## Figures and Tables

**Table 1:** Pathway enrichment analysis: List of significantly enriched pathways (false discovery rate (FDR) < 0.05).

Kegg Pathway	FDR	Coverage (%)	Associated Proteins
TCA cycle	8.10 × 10^−3^	13.33	ACLY, ACO2, IDH2, SDHA
Metabolic pathways	8.10 × 10^−3^	1.84	ACACA, ACLY, ACO2, AKR1B1, ALDH1A2, ALDH1B1, ALDH6A1, ASAH1, ATIC, CES1, COX5B, DCXR, EPRS1, GRHPR, HEXB, IDH2, NANS, NDUFS1, PGD, PRDX6, RPN1, SDHA, SORD
Lysosome	8.10 × 10^−3^	5.69	ASAH1, HEXB, LAMP1, LAMP2, NPC2, PSAP, SCARB2
Carbon metabolism	1.17 × 10^−2^	5.17	ACO2, ALDH6A1, CAT, IDH2, PGD, SDHA
Glutathione metabolism	2.20 × 10^−2^	8.00	GPX4, GSTP1, IDH2, PGD
IL-17 signalling pathway	2.20 × 10^−2^	5.43	HSP90AB1, LCN2, MAPK6, S100A8, S100A9
Glyoxylate and dicarboxylate metabolism	3.47 × 10^−2^	10.71	ACO2, CAT, GRHPR
Protein processing in ER	3.47 × 10^−2^	3.73	CRYAB, HSP90AB1, P4HB, RPN1, SEC61A1, UGGT1
Pentose and glucuronate interconversions	4.65 × 10^−2^	8.82	AKR1B1, DCXR, SORD

Proteins that were upregulated in patients with PCa in comparison to controls are labelled in green, while proteins labelled in red were downregulated in PCa. The abbreviations are listed in the abbreviation section.

**Table 2:** Shortlisted biological hallmarks that were found to be significantly enriched against Molecular Signatures Database (MSigDB) hallmark gene sets.

Hallmark	*p*-Value	Coverage (%)	Associated Proteins
MYC targets v1	1.64 × 10^−5^	5.00	EEF1B2, EPRS1, HSP90AB1, PA2G4, PCBP1, PSMB2, RPS5, SNRPD3, RACK1, HNRNPA3
Oxidative phosphorylation	5.15 × 10^−5^	4.50	ACO2, SLC25A6, COX5B, ECI1, GPX4, IDH2, ALDH6A1, NDUFS1, SDHA
Xenobiotic metabolism	5.15 × 10^−5^	4.50	ACO2, CA2, CAT, CES1, FBLN1, PGD, PDLIM5, DCXR, DHRS7
Fatty acid metabolism	5.51 × 10^−4^	4.43	ACO2, CA2, ECI1, HPGD, SDHA, GRHPR, PRDX6
Adipogenesis	1.92 × 10^−3^	3.50	ACLY, ACO2, CAT, COL4A1, GPX4, LAMA4, DHRS7, PRDX6
Protein secretion	2.22 × 10^−3^	5.21	AP2B1, ARCN1, ARF1, KRT18, LAMP2
Androgen response	2.41 × 10^−3^	4.95	HPGD, KRT8, PA2G4, SORD, PDLIM5
Oestrogen response late	6.88 × 10^−3^	3.00	CA2, FLNB, IDH2, S100A9, SORD, DCXR
Heme metabolism	6.88 × 10^−3^	3.00	CA1, CA2, CAT, LAMP2, ALDH6A1, SELENBP1
PI3K/AKT/mTOR signalling	1.60 × 10^−2^	3.81	ACACA, ARF1, UBE2N, YWHAB

Top 10 hallmarks selected based on the significance level (Benjamini–Hochberg adjusted *p*-value) are shown. Proteins that were upregulated in patients with PCa in comparison to controls are labelled in green, while proteins labelled in red were downregulated in PCa. The abbreviations are listed in the abbreviation section.

**Table 3:** List of differentially abundant proteins mapped to enriched pathways/hallmarks and associated with Gleason score (GS).

Protein Name	Symbol	Proteomics						Transcriptomics	Pathway/Hallmark
		Avg. Abundance PCa (±SD)	Avg. Abundance BPH (±SD)	Fold Change (PCa/BPH)	*p*-Value (MW)	Rho	*p*-Value (Spearman)	*p*-Value (ANOVA)	
Cytochrome c oxidase subunit 5B, mitochondrial	COX5B	35.57 (±46.07)	0.00 (±0.00)	Only in PCa	2.38 × 10^−2^	0.71	1.51 × 10^−3^	0.373	Metabolic pathways, Oxidative phosphorylation
Elongation factor 1-beta	EEF1B2	49.80 (±54.02)	0.00 (±0.00)	Only in PCa	5.62 × 10^−3^	0.52	3.12 × 10^−2^	0.173	MYC targets v1
Bifunctional glutamate/proline–tRNA ligase	EPRS1	113.27 (±111.41)	2.55 (±5.71)	44.39	1.08 × 10^−2^	0.56	1.88 × 10^−2^	<0.001	Metabolic pathways, MYC targets v1
Enoyl-CoA delta isomerase 1, mitochondrial	ECI1	83.71 (±144.87)	2.43 (±5.43)	34.46	4.25 × 10^−2^	0.55	2.08 × 10^−2^	<0.001	Oxidative phosphorylation, Fatty acid metabolism
PDZ and LIM domain protein 5	PDLIM5	49.56 (±95.10)	1.76 (±3.94)	28.15	1.60 × 10^−2^	0.51	3.79 × 10^−2^	<0.001	Xenobiotic metabolism, Androgen response
Methylmalonate-semialdehyde dehydrogenase (acylating), mitochondrial	ALDH6A1	320.75 (±466.95)	13.95 (±31.19)	22.99	9.02 × 10^−3^	0.56	2.01 × 10^−2^	<0.001	Metabolic pathways, Carbon metabolism, Oxidative phosphorylation, Heme metabolism
Protein transport protein Sec61 subunit alpha isoform 1	SEC61A1	30.88 (±32.34)	1.86 (±4.16)	16.59	2.46 × 10^−2^	0.75	5.71 × 10^−4^	<0.001	Protein processing in ER
Coatomer subunit delta	ARCN1	101.90 (±101.48)	10.27 (±10.20)	9.92	1.50 × 10^−2^	0.57	1.62 × 10^−2^	0.302	Protein secretion
Proliferation-associated protein 2G4	PA2G4	100.95 (±102.47)	17.45 (±24.51)	5.79	4.89 × 10^−2^	0.64	5.43 × 10^−3^	<0.001	MYC targets v1, Androgen response
Lysosome-associated membrane glycoprotein 1	LAMP1	240.92 (±193.22)	42.06 (±53.65)	5.73	2.29 × 10^−2^	0.58	1.43 × 10^−2^	0.036	Lysosome
Receptor of activated protein C kinase 1	RACK1	337.82 (±237.92)	65.47(±34.60)	5.16	6.10 × 10^−3^	0.67	3.38 × 10^−3^	<0.001	MYC targets v1
Glyoxylate reductase/hydroxypyruvate reductase	GRHPR	96.02 (±81.68)	22.89 (±16.11)	4.19	9.73 × 10^−3^	0.55	2.20 × 10^−2^	0.010	Metabolic pathways, Glyoxylate and dicarboxylate metabolism, Fatty acid metabolism
Protein disulphide-isomerase	P4HB	849.09 (±736.21)	205.58 (±45.05)	4.13	2.83 × 10^−2^	0.62	8.49 × 10^−3^	<0.001	Protein processing in ER
ADP-ribosylation factor 1	ARF1	274.51 (±227.49)	81.88 (±49.35)	3.35	3.44 × 10^−2^	0.57	1.78 × 10^−2^	<0.001	Protein secretion, PI3K/AKT/mTOR signalling
Dolichyl-diphosphooligosaccharide–protein glycosyltransferase subunit 1	RPN1	154.05 (±107.39)	50.38 (±29.21)	3.06	3.44 × 10^−2^	0.50	4.17 × 10^−2^	0.005	Metabolic pathways, Protein processing in ER
ADP/ATP translocase 3	SLC25A6	944.05 (±505.41)	359.42 (±146.11)	2.63	1.88 × 10^−2^	0.75	4.71 × 10^−4^	<0.001	Oxidative phosphorylation
Heat shock protein HSP 90-beta	HSP90AB1	1251.54 (±791.26)	482.75 (±165.56)	2.59	1.52 × 10^−2^	0.75	5.42 × 10^−4^	0.001	IL-17 signalling pathway, Protein processing in ER, MYC targets v1
Isocitrate dehydrogenase (NADP), mitochondrial	IDH2	546.27 (±279.70)	243.42 (±104.52)	2.24	2.83 × 10^−2^	0.57	1.72 × 10^−2^	0.017	TCA cycle, Metabolic pathways, Carbon metabolism, Glutathione metabolism, Oxidative phosphorylation, Oestrogen response late
14-3-3 protein beta/alpha	YWHAB	684.20 (±305.98)	324.49 (±159.83)	2.11	9.73 × 10^−3^	0.85	1.93 × 10^−5^	0.218	PI3K/AKT/mTOR signalling
Ubiquitin-conjugating enzyme E2 N	UBE2N	314.95 (±140.93)	159.39 (±58.37)	1.98	1.88 × 10^−2^	0.63	7.16 × 10^−3^	<0.001	PI3K/AKT/mTOR signalling
15-hydroxyprostaglandin dehydrogenase (NAD(+))	HPGD	37.40 (±37.39)	84.41 (±33.97)	0.44	3.16 × 10^−2^	−0.74	6.69 × 10^−4^	0.016	Fatty acid metabolism, Androgen response
Acetyl-CoA carboxylase 1	ACACA	9.93 (±20.78)	29.28 (±23.17)	0.34	4.09 × 10^−2^	0.60	1.16 × 10^−2^	<0.001	Metabolic pathways, PI3K/AKT/mTOR signalling
Aldehyde dehydrogenase X, mitochondrial	ALDH1B1	11.64 (±11.27)	40.54 (±37.31)	0.29	1.81 × 10^−2^	−0.49	4.38 × 10^−2^	<0.001	Metabolic pathways
Aldo-keto reductase family 1 member B1	AKR1B1	100.00 (±138.86)	377.89 (±231.11)	0.26	6.09 × 10^−3^	0.65	4.61 × 10^−3^	<0.001	Metabolic pathways, Pentose and glucuronate interconversions
Carbonic anhydrase 2	CA2	23.15 (±39.16)	191.60 (±176.21)	0.12	3.39 × 10^−3^	0.78	2.32 × 10^−4^	<0.001	Xenobiotic metabolism, Fatty acid metabolism, Oestrogen response late, Heme metabolism

Fold change (increase in green; decrease in red) calculated based on the average protein abundance in patients with PCa in comparison to average protein abundance in patients with BPH as well as unadjusted *p*-values (Mann Whitney test, MW) are provided. The results of the correlation analysis of protein abundance with GS are also given, including Spearman Rho correlation coefficient and relevant *p*-values. Association of mRNA abundance with disease progression retrieved from the Prostate Cancer Transcriptome Atlas is also presented. The abbreviations are listed in the abbreviations section.

## Supplementary Materials

The following are available online at https://www.mdpi.com/2075-4418/10/9/655/s1. **Table S1.** Clinical data: Baseline clinical data for patients with PCa and BPH are provided. **Table S2**. List of differentially abundant proteins: number of peptides belonging to protein, fold change, *p*-value (Mann Whitney test) and normalised protein abundance in individual samples are given. Fold change was calculated as a ratio between average protein abundance in patients with PCa and average protein abundance in patients with BPH.

## Abbreviations

ACACA	acetyl-CoA carboxylase 1
ACLY	ATP-citrate synthase
ACO2	aconitate hydratase, mitochondrial
ACTB	actin, cytoplasmic 1
ACTG2	actin, gamma-enteric smooth muscle
AKR1B1	aldo-keto reductase family 1 member B1
ALB	serum albumin
ALDH1A2	retinal dehydrogenase 2
ALDH1B1	aldehyde dehydrogenase X, mitochondrial
ALDH6A1	methylmalonate-semialdehyde dehydrogenase (acylating), mitochondrial
AP2B1	AP-2 complex subunit beta
AR	androgen receptor
ARCN1	coatomer subunit delta
ARF1	ADP-ribosylation factor 1
ASAH1	acid ceramidase
ATIC	bifunctional purine biosynthesis protein ATIC
BPH	benign prostatic hyperplasia
CA1	carbonic anhydrase 1
CA2	carbonic anhydrase 2
CAT	catalase
CES1	liver carboxylesterase 1
COL4A1	collagen alpha-1(IV) chain
COL6A3	collagen alpha-3(VI) chain
CORO1B	coronin-1B
COX5B	cytochrome c oxidase subunit 5B, mitochondrial
CRPC	castration-resistant prostate cancer
CRYAB	alpha-crystallin B chain
CSRP2	cysteine and glycine-rich protein 2
DCXR	L-xylulose reductase
DES	desmin
DHRS7	dehydrogenase/reductase SDR family member 7
ECI1	enoyl-CoA delta isomerase 1, mitochondrial
EEF1B2	elongation factor 1-beta
EPRS1	bifunctional glutamate/proline–tRNA ligase
ER	endoplasmic reticulum
FBLN1	fibulin-1
FDR	false discover rate
FLNA	filamin-A
FLNB	filamin-B
GPX4	phospholipid hydroperoxide glutathione peroxidase
GRHPR	glyoxylate reductase/hydroxypyruvate reductase
GS	Gleason score
GSTP1	glutathione S-transferase P
HBB	haemoglobin subunit beta
HCD	higher-energy collisional dissociation
HEXB	beta-hexosaminidase subunit beta
HIST1H2AH	histone H2A type 1-H
HNRNPA3	heterogeneous nuclear ribonucleoprotein A3
HPGD	15-hydroxyprostaglandin dehydrogenase (NAD(+))
HSP90/HSP90AB1	heat shock protein HSP 90-beta
IDH2	isocitrate dehydrogenase (NADP), mitochondrial
IL-17	interleukin 17
ILF3	interleukin enhancer-binding factor 3
IR	interquartile range
IRE1α	inositol requiring-enzyme 1 alpha
Kegg	Kyoto encyclopedia of genes and genomes
KRT18	keratin, type I cytoskeletal 18
KRT8	keratin, type II cytoskeletal 8
LAMA4	laminin subunit alpha-4
LAMP1	lysosome-associated membrane glycoprotein 1
LAMP2	lysosome-associated membrane glycoprotein 2
LC-MS/MS	liquid chromatography coupled to tandem mass spectrometry
LCN2	neutrophil gelatinase-associated lipocalin
MAPK6	mitogen-activated protein kinase 6
mCRPC	metastatic castration-resistant prostate cancer
MSigDB	Molecular Signatures Database
MW	Mann Whitney test
MYC	Myc proto-oncogene
MYH11	myosin-11
NANS	sialic acid synthase
NDUFS1	NADH-ubiquinone oxidoreductase 75 kDa subunit, mitochondrial
NPC2	NPC intracellular cholesterol transporter 2
P4HB	protein disulphide-isomerase
PA2G4	proliferation-associated protein 2G4
PCa	prostate cancer
PCBP1	poly(rC)-binding protein 1
PDLIM5	PDZ and LIM domain protein 5
PGD	6-phosphogluconate dehydrogenase, decarboxylating
PRDX6	peroxiredoxin-6
PSA	prostate specific antigen
PSAP	prosaposin
PSMB2	proteasome subunit beta type-2
RACK1	receptor of activated protein C kinase 1
RPN1	dolichyl-diphosphooligosaccharide-protein glycosyltransferase subunit 1
RPS5	ribosomal protein S5
S100A8	protein S100-A8
S100A9	protein S100-A9
SCARB2	lysosome membrane protein 2
SDHA	succinate dehydrogenase (ubiquinone) flavoprotein subunit, mitochondrial
SDS-PAGE	sodium dodecyl sulphate–polyacrylamide gel electrophoresis
SEC61A1	protein transport protein Sec61 alpha isoform 1
SELENBP1	methanethiol oxidase
SLC25A6	ADP/ATP translocase 3
SNRPD3	small nuclear ribonucleoprotein Sm D3
SORD	sorbitol dehydrogenase
TAGLN	transgelin
TCA cycle	tricarboxylic acid cycle
UBE2N	ubiquitin-conjugating enzyme E2 N
UGGT1	UDP-glucose:glycoprotein glucosyltransferase 1
XBP1	X-box-binding protein 1
YWHAB	14-3-3 protein beta/alpha
